# Properties and molecular identity of NMDA receptors at synaptic and non-synaptic inputs in cerebellar molecular layer interneurons

**DOI:** 10.3389/fnsyn.2015.00001

**Published:** 2015-02-20

**Authors:** Céline Bidoret, Guy Bouvier, Annick Ayon, Germán Szapiro, Mariano Casado

**Affiliations:** Département de Biologie, Ecole Normale Supérieure, Institut de Biologie de l’ENS (IBENS), and Inserm U1024, and CNRS UMR 8197Paris, France

**Keywords:** cerebellum, NMDA receptors, interneuron, plasticity, parallel fibres, climbing fibres

## Abstract

N-methyl-D-aspartate receptors (NMDARs) in cerebellar molecular layer interneurons (MLIs) are expressed and activated in unusual ways: at parallel fibre (PF) synapses they are only recruited by repetitive stimuli, suggesting an extrasynaptic location, whereas their activation by climbing fibre is purely mediated by spillover. NMDARs are thought to play an important role in plasticity at different levels of the cerebellar circuitry. Evaluation of the location, functional properties and physiological roles of NMDARs will be facilitated by knowledge of the NMDAR isoforms recruited. Here we show that MLI-NMDARs activated by both PF and climbing fibre inputs have similar kinetics and contain GluN2B but not GluN2A subunits. On the other hand, no evidence was found of functional NMDARs in the axons of MLIs. At the PF-Purkinje cell (PF-PC) synapse, the activation of GluN2A-containing NMDARs has been shown to be necessary for the induction of long-term depression (LTD). Our results therefore provide a clear distinction between the NMDARs located on MLIs and those involved in plasticity at PF-PC synapses.

## Introduction

N-methyl-D-aspartate receptors (NMDARs) are normally composed of two copies of GluN1 and two of GluN2 subunits. GluN2 is coded by four different genes (GluN2A, 2B, 2C and 2D). The subunit composition defines the biophysical, pharmacological and signaling properties of NMDARs. In particular, during NMDAR-dependent spike timing-dependent plasticity, the receptor kinetics, endowed by the GluN2 subtypes, may participate in setting a time window for potentiation and depression (Markram et al., 1997; Bi and Poo, 1998; Sjöström et al., 2001). Cerebellar molecular layer interneurons (MLIs) express NMDARs but a detailed functional characterization of the isoforms expressed is lacking. *In situ* hybridization studies have shown the expression of GluN1 and GluN2D subunits (Akazawa et al., 1994; Watanabe et al., 1994) and immunohistological studies reveal the expression of GluN1 and GluN2A/B subunits (with a non-segregating antibody; Petralia et al., 1994).

MLIs receive excitatory inputs from parallel fibres (PFs) and climbing fibres (CFs). Postsynaptic NMDARs at the PF to interneuron synapse (PF-MLI) are located extrasynaptically. As a consequence, single PF stimulation results in AMPA-only excitatory postsynaptic currents (EPSCs) and a NMDA component is apparent only after repetitive or strong stimulation (Carter and Regehr, 2000; Clark and Cull-Candy, 2002; Nahir and Jahr, 2013). CFs signal to interneurons via spillover recruiting AMPARs and NMDARs (Szapiro and Barbour, 2007; Coddington et al., 2013). Crosstalk between PF and CF inputs on MLIs has been proposed to be a key element of cerebellar function (Ekerot and Jörntell, 2001) but little is known on the nature of this crosstalk.

MLIs synapse onto each other as well as onto Purkinje cells (PCs) and Golgi cells. The presence of axonal NMDARs in MLIs has been mainly inferred from recordings of miniature inhibitory postsynaptic currents (mIPSCs). In particular, bath application of NMDA has been reported to increase the frequency of incoming mIPSCs on both MLIs and PCs (Glitsch and Marty, 1999; Duguid and Smart, 2004; Huang and Bordey, 2004; Glitsch, 2008; Rossi et al., 2012). Alternatively, this effect has been attributed to the activation of dendritic NMDARs on MLIs (Christie and Jahr, 2008; Pugh and Jahr, 2011).

NMDARs play an important role in plasticity at different levels of the cerebellar molecular layer circuitry. At the PF-MLI synapse, NMDARs are involved in long-term potentiation (LTP; Rancillac and Crépel, 2004; Smith and Otis, 2005). *In vivo*, potentiation at this synapse requires the activation of CFs (Jörntell and Ekerot, 2002, 2003). Therefore, a crosstalk between NMDARs of PF and CF inputs would have important consequences for MLI functions. Besides, at the PF-PC synapse, NMDA-dependent long-term depression (LTD) can be induced by simultaneous activity of PFs and CFs (Ito and Kano, 1982; Casado et al., 2002). Significantly, fast deactivating receptors containing the GluN2A subunit have been shown to be required for PF-PC LTD (Bidoret et al., 2009). However, the location of these receptors is controversial; they have been proposed to be located on PFs (Casado et al., 2002; Bidoret et al., 2009), on MLI dendrites (Wang et al., 2014) or on MLI axons (Shin and Linden, 2005).

A detailed knowledge of the NMDAR subunits activated in MLIs under physiological conditions would help to identify the specific actors involved in synaptic plasticity at different points of the cerebellar ML circuitry. Here we studied the pharmacological and kinetic properties of NMDARs in MLIs of juvenile rats. First, we could not find any effect of NMDA application on mIPSCs recorded in both MLIs and PCs, suggesting the absence of functional NMDARs on MLI axons at this developmental stage. Then, we found that both PFs and CFs recruit mostly GluN2B-containing NMDARs with homogeneous kinetics, and we confirmed the presence of GluN2B subunits on MLI somatodendritic compartment by immunohistochemistry. The relatively fast kinetics of these receptors may allow a rather sharp time window for input integration in MLIs. Moreover, this characterization establishes a distinction between the NMDARs present in MLIs and those involved in plasticity at PF-PC synapses.

## Materials and methods

### Electrophysiology

Animal experimentation methods complied with French and European regulations and were authorized by the “Direction Départementale des Services Vétérinaires de Paris” according to the project submitted by the “Cerebellum team” at ENS, approval number 75-500-Renouvellement. Male Wistar rats (P18–P24) were killed by decapitation and the cerebellum rapidly dissected into a Kgluconate-based solution (mM): 130 KGluconate, 15 KCl, 0.2 EGTA, 20 HEPES, 25 D-glucose, bubbled with 95% O_2_/5% CO_2_, (pH 7.4) supplemented with 50 μM D-APV. Parasagittal (MLI recordings, with no distinction between basket and stellate cells) or transverse (PC recordings) cerebellar acute slices (300–350 μm) were cut in the same solution using a Microm HM650V slicer. Slices recovered at least for 60 min in extracellular saline at 33°C, containing (mM): 125 NaCl, 2.5 KCl, 2 CaCl_2_, 1 MgCl_2_, 1.25 NaH_2_PO_4_, 26 NaHCO_3_, 25 D-glucose; bubbled with 95% O_2_/5% CO_2_ (pH 7.4). The recording chamber was perfused at a rate of 2–3 ml/min with the same solution supplemented with 10 mM tricine (a Zn^2+^ ion buffer, Paoletti et al., 1997). Experiments were performed at 32°C (Single Channel Heater Controller, Warner Instruments, Holliston, MA).

Patch pipettes had resistances of 2–4 MΩ and 4–6 MΩ for PCs and MLIs respectively. Cells were voltage-clamped at −70 mV in the whole-cell configuration. Series resistance was held between 4 and 10 MΩ, compensated with settings of 70% for PCs and between 10 and 20 MΩ, compensated with settings of 30–70%, for MLIs. pCLAMP8 software (Molecular Dynamics) was used for data acquisition and analysis. Recordings were filtered at 1 kHz and digitized at 50 kHz.

For EPSC recordings the internal solution contained (mM): 120 KGluconate, 0.5 K_3_Citrate, 0.5 L(-)Malic acid, 0.008 Oxaloacetic acid, 0.18 α-Ketoglutaric acid, 0.2 Pyridoxal-5′-phosphate, 5 L-Alanine, 0.15 Pyruvic acid, 15 L-Glutamine, 4 L-Asparagine, 1 L-Glutathione reduced, 0.5 NAD+, 5 Phosp-hocreatine-K_2_, 10 HEPES, 0.1 K_3_EGTA, 4 KCl, 2.2 K_2_Phosphate, 3.5 NaAcetate, 0.05 CaCl_2_, 2.1 Mg-ATP, 0.4 Na_2_-GTP, 1.4 Na_2_-ATP, pH 7.3 with KOH. Gabazine 25 μM and muscimol 5 μM were added to the external solution. The reason to add gabazine and muscimol at the same time is that, when recording NMDA mediated responses in MLIs two signals can interfere with the recordings: IPSCs and spikelets due to excitation of electrically coupled MLIs. Gabazine alone will block IPSCs, but increase interneuron excitability. IPSCs are still blocked when muscimol is added to gabazine (all GABA-binding sites are occupied), but muscimol is able to compete with gabazine and activate a tonic hyperpolarising conductance in MLIs which in turn prevents excitation of coupled interneurons. EPSCs were evoked by stimulating PFs and CFs extracellularly by means of a glass pipette (tip diameters 8–12 μm and 2–4 μm respectively) filled with HEPES-buffered saline.

For inhibitory miniature event recordings, the internal solution contained (mM): 145 CsCl, 10 HEPES, 1 EGTA, 5 MgCl_2_, 0.1 CaCl_2_, 4 Na_2_-ATP, 0.4 Na_2_-GTP (pH 7.3 with CsOH). For MLI recordings, 1 mM MK801 was added to the internal solution to block postsynaptic NMDA receptors. 10 μM NBQX, 1 μM CGP55845, 200 nM DPCPX and 200 nM TTX were added to the bath to block AMPA, GABA-B, adenosine A1 receptors and sodium channels respectively.

### Chemicals

Tricine 10 mM was used for Zn^2+^ buffering to obtain 300 nM free Zn^2+^ from 60 μM total ZnCl_2_ as described by Paoletti et al. (1997). Picrotoxin, CGP55845 and DPCPX were purchased from Tocris Cookson (Bristol, UK), NBQX, D-APV and TTX were from Ascent Scientific (Weston-Super-Mare, UK). Ro25-6981 was from Roche (Basel, Switzerland). All the other chemicals were purchased from Sigma (St. Louis, MO).

### Immunohistochemistry

For fluorescence immunohistochemistry, MLIs in 300 μm slices were filled with 2 mM neurobiotin (Thermo Scientific), by means of the patch pipette. Slices were fixed in 4% paraformaldehyde in PBS (PFA) for 20 min at room temperature for GluN2A labelling (10 min at pH 7 plus 10 min at pH 9), and in 4% PFA for 70 min at room temperature for GluN2B labelling (10 min at pH 7 plus 60 min at pH 9). Residual PFA was inactivated with 50 mM NH_4_Cl in PBS. Slices were included in gelatin (G2500; Sigma) to be resliced in 70 μm sections. Section tissue pre-treatment for GluN2A labelling consisted of 0.5% triton-X-100 incubation, in a solution of 100 mM Tris, 150 mM NaCl, 0.5% blocking reagent (FP1020; Perkin Elmer) (TNB), for 2 h at 37°C. For GluN2B labelling, pre-treatment consisted of heating in a 25 mM glycine-HCl pH 3 solution for 30 min at 80°C; sections were then incubated in TNB with 0.2% triton-X-100 for 1 h at room temperature. GluN2A and GluN2B labelling were performed by incubating sections, 20 h at 4°C, first with mouse monoclonal antibodies (GluN2A 7.5 μg/ml; MAB5216, Chemicon, GluN2B 1 μg/ml; 610416, BD Transduction Laboratories; Specificity checked in Bidoret et al., 2009) then with anti-mouse IgG coupled to Alexa 555 (4 μg/ml; Molecular Probes). Neurobiotin was revealed with streptavidin coupled to Alexa 488 (1/200) (Life Technologies). Stained tissue was observed in a confocal microscope (SP5, Leica) with a 63X objective and images acquired at voxel size 46 nm, 46 nm, 210 nm (x,y,z). Presence of staining on filled cells was evaluated using a 3D Costes’ randomization algorithm implemented in the JaCoP plugin for ImageJ (Costes et al., 2004; Bolte and Cordelières, 2006).

Pre-embedding immuno electron microscopy in cerebellar tissue from a 24-day-old Wistar rat was performed as described in Bidoret et al. (2009). Micrographs were analyzed using ImageJ software. MLIs were recognized by their soma and classical nuclear invaginations. PF synapses onto MLI somata were identified by their postsynaptic densities. The quantification of the peroxidase labelling was done on ~17500 μm^2^ for each section, in lobules 5 to 7 of the cerebellar cortex.

### Statistics

Data are reported as mean ± s.d. Significance was tested using the nonparametric Wilcoxon test (two-tailed) in GNU R (R Foundation for Statistical Computing).

## Results

We first characterized the molecular nature of postsynaptic NMDARs on MLIs (with no distinction between stellate and basket cells). Pharmacological compounds allowing a distinction between GluN2A and GluN2B-containing receptors have been extensively described: 300 nM free Zn^2+^ blocks receptors composed of GluN2A with no effect on those composed of GluN2B or GluN2C subunits (Paoletti et al., 1997; Rachline et al., 2005; Bidoret et al., 2009). Conversely, 300 nM Ro25-6981, an analogue of ifenprodil, blocks receptors containing GluN2B subunits with no effect on GluN2A or GluN2C containing receptors (Fischer et al., 1997; Bidoret et al., 2009).

**Figure 1:**
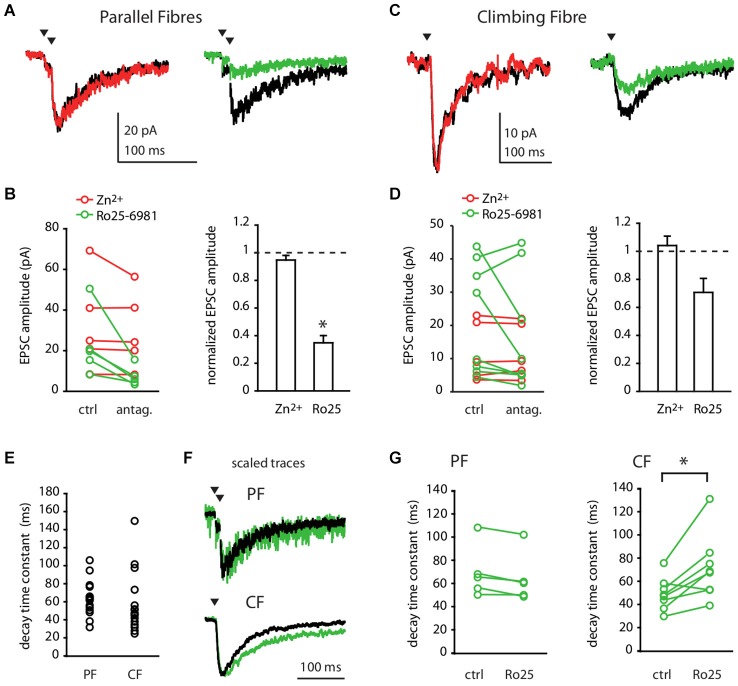
**Parallel Fibre and Climbing Fibre inputs activate GluN2B-containing NMDA receptors in Molecular Layer interneurons. (A,B)** PF- or **(C,D)** CF-evoked NMDA currents in MLIs in control conditions (black) or in the presence of 300 nM free Zn^2+^ (red) or 300 nM Ro25-6981 (green). **(A,C)** Representative recordings (average of 30–50 sweeps). **(B,D)** Left panels: NMDA EPSC amplitude before (ctrl) and after (antag.) bath application of Zn^2+^ (red) or Ro25-6981 (green) for each cell. Right panels: Mean normalized NMDA-EPSC amplitude after Zn^2+^ or Ro25-6981.*p < 0.05. **(E)** Decay time constants of PF- or CF-evoked NMDA EPSCs in control conditions. **(F,G)** Decay time constants of CF- but not of PF-evoked NMDA EPSCs are slowed by Ro25-6981. **(F)** Scaled traces of representative recordings (average of 30–50 sweeps) in control conditions (black) and after 300 nM Ro25-6981 (green). For PF input, same cell as in **(A)**. For CF input, different cell from **(C)**. **(G)** PF (left) and CF (right) -evoked NMDA EPSC decay time constant before and after bath application of Ro25-6981 for each cell. *p < 0.05.

At PF-MLI synapses, single stimuli do not activate NMDA receptors; only a train of stimuli produces a detectable NMDAR current (Clark and Cull-Candy, 2002). Therefore, we recorded NMDA currents in MLIs in response to a pair of PF stimulations at 100 Hz, in the absence of extracellular magnesium and in the presence of NBQX, an AMPA receptor antagonist. Figure 1 shows the effect of Zn^2+^ and Ro25-6981 on the NMDA response. At the PF-MLI synapse, NMDA currents were insensitive to Zn^2+^ (Figures 1A,B; 32.87 ± 23.41 pA before vs. 30.00 ± 18.88 pA after treatment, *n* = 5, *p* = 0.19) but were significantly reduced by Ro25-6981 (Figures 1A,B; 30.45 ± 23.43 pA before vs. 10.55 ± 8.97 pA after treatment, *n* = 6, *p* = 0.03). When measurable, PF-MLI NMDA EPSC decay kinetics (Figure 1E, 69.52 ± 27.03 ms, *n* = 17) were only slightly affected by Ro25-6981 treatment (Figures 1F,G; 68.51 ± 22.78 ms before vs. 63.46 ± 21.81 ms after treatment, *n* = 5, *p* = 0.06). These currents were fully blocked by D-APV (0.46 ± 0.16 pA, *n* = 5 for the Ro25-6981 series; 0.55 ± 0.31 pA, *n* = 4 for the Zn^2+^series). Similar pharmacological profiles were obtained in experiments performed at room temperature with 4-stimulation trains at 100 Hz (not shown). These results show that MLI NMDARs activated by PF inputs are sensitive to GluN2B- but not to GluN2A-specific pharmacology.

We next characterized the NMDARs at the CF-MLI connection. We recorded NMDA currents evoked in MLIs in response to a single CF stimulation, in the absence of extracellular magnesium and in the presence of NBQX. The evoked NMDA currents were insensitive to Zn^2+^ (Figures 1C,D; 12.69 ± 7.86 pA before vs. 13.46 ± 8.67 pA after treatment, *p* = 1, *n* = 6). On the other hand, Ro25-6981 had heterogeneous effects on the NMDA current amplitude. Even if the mean outcome was a 23% inhibition of the current, Ro25-6981 effect in individual experiments ranged from strong inhibition to little or no apparent effect (Figures 1C,D; 22.07 ± 16.6 pA before vs. 16.96 ± 17.28 pA after treatment, *p* = 0.31, *n* = 8). Like PF-NMDA EPSCs, CF-NMDA EPSCs displayed relatively fast decay kinetics (Figure 1E; 58.84 ± 33.21 ms, *n* = 16; PF vs. CF *p* = 0.06). Interestingly, the decay time of the CF-NMDA current was significantly slowed by Ro25-6981 in most recordings (Figures 1F,H; 48.4 ± 13.9 ms before vs. 70.6 ± 27.8 ms after treatment, *p* = 0.01, *n* = 8). This slowing is consistent with the mode of action of Ro25-6981 which has been reported to increase the affinity of GluN2B-containing NMDARs for glutamate and thus to potentiate and prolong the decay time of NMDA currents at low glutamate concentrations (Kew et al., 1996; Fischer et al., 1997). The increase in the affinity of GluN2B-containing receptors for glutamate at low concentrations of the agonist would also account for the heterogeneous effect of Ro25-6981 on the amplitude of the CF NMDA responses. In a spillover connection, as it is the case for the CF-MLI connection, the distance between the release site and the postsynaptic receptors is variable. Thus, the effective concentration of glutamate reaching receptors will vary among different CF-MLI connections. This would explain the variability observed, most likely due to an interplay between the antagonist and potentiating effects of Ro25-6981. All these currents were D-APV sensitive (0.02 ± 0.79 pA, *n* = 5 for the Zn^2+^ series; 0.92 ± 0.76 pA, *n* = 3 for the Ro25-6981 series). Together, these results show that MLI NMDARs activated by CF inputs present a variable degree of sensitivity to GluN2B-specific antagonist but are consistently insensitive to GluN2A-specific pharmacology.

**Figure 2:**
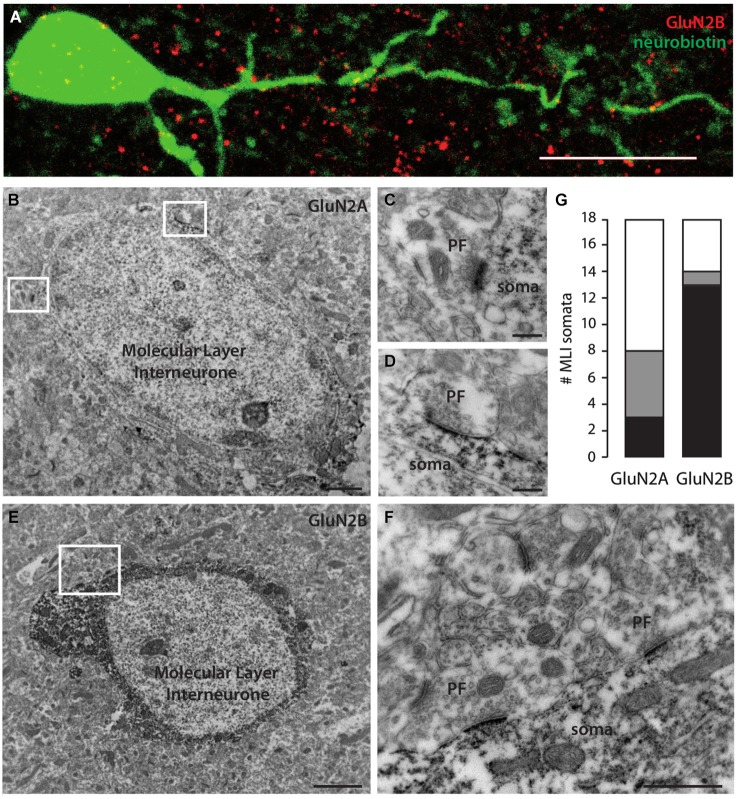
**Molecular Layer interneurons express mostly GluN2B-containing NMDA receptors in their somatodendritic compartment. (A)** Immunostaining showing GluN2B (red) colocalization with a neurobiotin-filled MLI somatodendritic compartment (green). Sum of 30 optical slices in a stack. Scale bar = 10 μm. **(B,E)** MLI somata immunostained for GluN2A **(B)** or GluN2B **(E)**. Scale bars = 2 μm. **(C,D)** Examples of unstained PF synapses onto GluN2A-stained MLI soma extended from **(B)**. Scale bars = 200 nm. **(F)** Examples of unstained PF synapses onto GluN2B-stained MLI soma extended from **(E)**. Scale bar = 500 nm. **(G)** Quantification of the number of MLI somata faintly (gray) or strongly (black) stained for GluN2A or GluN2B in 17500 μm^2^ areas.

To obtain independent corroboration of these results, we performed immunohistochemistry with antibodies directed against GluN2A and GluN2B subunits. We first filled MLIs with neurobiotin through patch pipettes; further revelation with the streptavidin complex allowed quantification of the colocalization with immunostaining. Both antibodies produced punctate staining in the molecular layer. GluN2B staining showed significant colocalization with the dendritic arborisation of MLIs (Figure 2A, Pearson’s coefficient of 0.085 vs. −0.001 ± 0.003 after Costes’ randomization for an analyzed dendritic volume of 284 μm^3^ in a total of 2290 μm^3^). GluN2A staining was less conclusive but a Pearson’s coefficient of 0.016 vs. 0.000 ± 0.001 was still indicative of a lower but significant coincidence with cell filling (for an analyzed dendritic volume of 13 μm^3^ in a total of 2157 μm^3^).

To gain further insight into the distribution of NMDAR subunits, we performed electron immunohistochemistry. To eliminate possible signals arising from Golgi cell apical dendrites, we counted the somata of MLIs labeled for GluN2A or GluN2B. In tissue chunks (17500 μm^2^ studied area) stained with GluN2A antibody, 8 MLIs out of 18 were positive. The staining was often faint (5/8; Figures 2B,G). Interestingly, excitatory PF varicosities on MLI somata were negative for GluN2A (Figures 2C,D), in contrast to previously described GluN2A positive PF varicosities on PC spines (Bidoret et al., 2009). In tissue stained with GluN2B antibodies, 14 MLIs out of 18 were positive. Staining was almost systematically strong (13/14; Figures 2E,G). Again, PF varicosities on MLI somata were negative for GluN2B (Figure 2F). These results are consistent with the dominant expression of GluN2B over GluN2A subunits in the somatodendritic compartment of MLIs shown above in functional experiments (Figure 1).

**Figure 3:**
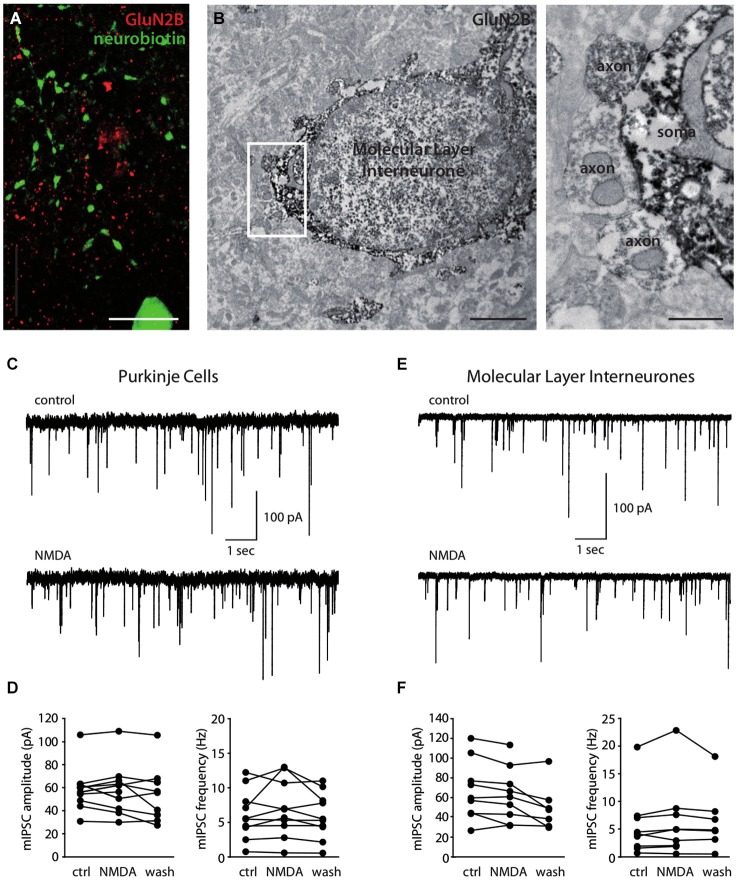
**Molecular Layer interneurons do not express functional axonal NMDA receptors. (A)** Immunostaining showing the absence of GluN2B (red) colocalization with a neurobiotin-filled MLI axon (green). Sum of 56 optical slices in a stack. Scale bar = 10 μm. **(B)** Immunostaining for GluN2B showing GluN2B-stained putative inhibitory synapses (axon) on a GluN2B-stained MLI soma. Synapses are extended in the right panel. Scale bars: left panel 2 μm, right panel 0.5 μm. **(C–F)** Miniature IPSCs recorded in PCs **(C,D)** or in MLIs **(E,F)** in control conditions or in the presence of 15 μM **(C,D)** or 30 μM **(E,F)** NMDA. **(C,E)** Representative recordings. **(D,F)** Amplitude and frequency of mIPSCs for each cell before (ctrl), during (NMDA) or after (wash) bath-application of NMDA.

We finally explored the expression of NMDARs on axons of MLIs. First, we searched for the expression of subunits at the ultrastructural level. Over 18 MLI somata studied, 7 putative axosomatic inhibitory synapses displaying presynaptic GluN2B staining could be identified after electron immunohistochemistry (Figure 3B). Consistent with this rare staining, comparison between GluN2B staining after fluorescence immunohistochemistry and morphologically identified MLI axons filled with neurobiotin was suggestive of exclusion (Pearson’s coefficient of −0.006 vs. 0.001 ± 0.001 after Costes’ randomization, for an analyzed axonal volume of 15 μm^3^ in a total of 3375 μm^3^; Figure 3A). In addition, no presynaptic GluN2A staining could be observed in inhibitory terminals (not shown). In conclusion, we found little or no expression of GluN2A- or GluN2B-containing NMDARs in MLI axons. However, we cannot exclude the expression of other subunits, in particular because NMDA has been shown to increase the frequency of mIPSCs recorded in MLIs and PCs (Glitsch and Marty, 1999; Duguid and Smart, 2004; Huang and Bordey, 2004; Glitsch, 2008), an effect generally attributed to presynaptic phenomena. To explore this possibility, we measured the effect of NMDA applications on the mIPSCs recorded in PCs in experimental conditions matching those of our previous experiments. In the presence of 1 mM Mg^2+^, 15 μM NMDA bath application did not elicit detectable changes in the amplitude or frequency of the mIPSCs (Figures 3C,D, 99.2 ± 11.2% and 112.0 ± 30.7% of control values for amplitude and frequency, respectively, *p* = 0.84 and *p* = 0.37, *n* = 10). Likewise, we measured the effects of NMDA applications on the mIPSCs recorded in MLIs. Again, 30 μM NMDA application produced no detectable changes in mIPSC amplitude or frequency (Figures 3E,F, 94.95 ± 12.90% and 106.73 ± 20.42% of control values for amplitude and frequency respectively, *p* = 0.80 and *p* = 0.10, *n* = 9). Together, these results suggest that, at least in juvenile rats under the present experimental conditions, MLI axons are devoid of functional NMDARs.

## Discussion

Integrative properties of synaptic inputs and synaptic plasticity depend strongly on the biophysical features of NMDARs. Differences in these properties amongst different subtypes of NMDARs may result in different learning rules. Here we have performed functional, pharmacological and immunohistochemical characterization of NMDARs in cerebellar MLIs. These neurons have two excitatory inputs, PFs and CFs, which convey sensory and motor command information to the cerebellar cortex (Eccles et al., 1967; Ito, 1984). By combining subunit-specific pharmacology and electrophysiology we showed that GluN2B-containing NMDARs were activated by PFs and CFs. In contrast, we found no evidence for functional GluN2A-containing receptors. The presence of GluN2B was confirmed by immunohistochemistry at both optical and electron microscopy levels, although the presence of a low level of GluN2A expression was also detected.

The decay time constant of the NMDA components of both PF (69.52.0 ± 27.03 ms) and CF inputs (58.84 ± 33.21 ms) is different from those of recombinant receptors at the same temperature (28.6 ± 4.7 ms, 193.2 ± 14.9 ms, 217.6 ± 37.6 ms for heterodimeric receptors composed of GluN1a plus GluN2A, GluN2B or GluN2C respectively, Bidoret et al., 2009). A first interpretation would be that somatodendritic NMDARs in MLIs are not heterodimers merely formed by GluN1 and GluN2B. Intermediate kinetics between GluN2A and GluN2B receptors could be due to the presence of heterotrimers formed by GluN1, GluN2A and GluN2B subunits (Hansen et al., 2014). This would be consistent with the small fraction of GluN2A staining observed in immunohistochemistry experiments. However, such heterotrimeric receptors should be sensitive to zinc (Hatton and Paoletti, 2005; Hansen et al., 2014) and a treatment with the antagonist Ro25-6981 should accelerate the decay, revealing the presence of GluN2A subunits (Hansen et al., 2014). We do not observe such effects, indicating that the GluN2A subunit is not present in the recorded receptors. *In situ* hybridization studies have also suggested the presence of GluN2D in MLIs (Akazawa et al., 1994; Watanabe et al., 1994). Although we couldn’t test the presence of the GluN2D subunit directly because of no specific pharmacology (Paoletti and Neyton, 2007), EPSC kinetics for both inputs were incompatible with GluN1-GluN2D heterodimers which decay in more than 1 s (at room temperature, Vicini et al., 1998; Misra et al., 2000). An alternative explanation would involve GluN1 splice variants. The kinetics of recombinant receptors reported in Bidoret et al. (2009) were obtained upon expression of GluN1a together with the different GluN2 subunits. However, when combined with GluN2B, the GluN1b splice variant accelerates 4.4 times the decay kinetics of the receptors (Rumbaugh et al., 2000). This would result in a decay time constant of about 44 ms for GluN1b + GluN2B combinations, in the range of the kinetics observed in Figure 1. Significantly, GluN1b is expressed in the cerebellar cortex (Prybylowski et al., 2000) and in particular, MLIs express predominantly this splice variant (Laurie and Seeburg, 1994). Interestingly, such a GluN1b + GluN2B combination confers intermediate kinetics between GluN1a + GluN2A and GluN1a + GluN2B receptors, defining therefore an intermediate window for synaptic integration by NMDARs.

The overall pharmacological and kinetic properties of the NMDAR components of PF and CF inputs are similar, although their sensitivity to the GluN2B antagonist is slightly different. Ro25-6981 significantly decreased the amplitude of the PF-MLI NMDA responses. In contrast, Ro25-6981 effect on the amplitude of the CF-MLI NMDA current was heterogeneous between individual experiments although the decay time was almost systematically slowed. The variability of the effect of Ro25-6981 on CF inputs can be explained by its mode of action: together with its antagonistic effect, this compound increases the affinity of GluN2B-containing receptors for glutamate. This results in a systematic slowing of the response (that reflects glutamate unbinding) and in potentiation only for receptors reached by low glutamate concentrations (Kew et al., 1996; Fischer et al., 1997). This second effect may be variable depending on the distance between release sites and postsynaptic receptors. Such heterogeneity is therefore expected for the pure spillover CF-MLI connection. In conclusion, we show that PFs and CFs, the sole excitatory inputs to MLIs, activate GluN2B- but not GluN2A-containing NMDA receptors. Whether both inputs activate at least partially the same population of receptors remains an open question.

Conflicting data have been published on the existence of presynaptic NMDA receptors in MLIs. Evidence for the presence of such receptors arises from different sets of studies. First, bath application of NMDA has been shown to increase mIPSC frequency in both MLIs and PCs (Glitsch and Marty, 1999; Huang and Bordey, 2004; Glitsch, 2008). Second, depolarization of PCs can give rise to a transient increase in mIPSC frequency that is sensitive to NMDAR antagonists (Duguid and Smart, 2004). More recently, 2-photon calcium imaging experiments combined with local uncaging of a photosensitive precursor of glutamate reported spots of calcium entry through NMDARs in the axons of MLIs (Rossi et al., 2012). In contrast, the group of Jahr (Christie and Jahr, 2008; Pugh and Jahr, 2011) was unable to detect direct activation of NMDARs on MLI axons. Rather, they showed that the effects of NMDA on mIPSCs could be explained from calcium increases through axonal voltage-dependent calcium channels after NMDAR-mediated somatodendritic depolarization (Christie and Jahr, 2008).

Our electrophysiological data do not provide evidence of functional NMDARs in the axons of juvenile rat MLIs. Bath-applied NMDA was ineffective in increasing neither the frequency nor the amplitude of mIPSCs on PCs or on MLIs, unlike some above mentioned studies. Interestingly, our electron microscopy data show the presence of GluN2B subunits on the presynaptic terminals of some axosomatic inhibitory contacts among MLIs. Nevertheless, such contacts may represent a minority of the inhibitory inputs on a given cell and their integrity on a patched cell may be compromised. Conflicting data from different laboratories may arise from differences in experimental conditions. In particular, the presence of axonal receptors could be sharply dependent on the stage of development and because granule cell migration in the rodent cerebellum is only completed at P17-P18 (Miale and Sidman, 1961), cells recorded before this stage of development should be heterogeneous. Studies supporting the presence of receptors have been mostly performed in developing rodents: rats aged P12 to P14 (Glitsch and Marty, 1999), P13 to P16 (Glitsch, 2008), P6 to P8 and P11 to P14 (Duguid and Smart, 2004), or mice aged P10 to P20 (Huang and Bordey, 2004) and P12 to P16 (Rossi et al., 2012). The group of Jahr used P8 to P18 (Pugh and Jahr, 2011) and P15 to P20 rats (Christie and Jahr, 2008). As for previous studies (Casado et al., 2000, 2002; Bidoret et al., 2009), we used rats aged P18 to P24 to make this study suitable for comparison with our previously published data and in particular to compare NMDARs in MLIs with those involved in PF-PC LTD induction (see below). In these conditions, our results do not support the presence of functional NMDARs on MLI axons from juvenile rats.

LTD at the PF-PC synapse depends on NMDAR activation (Casado et al., 2002; Shin and Linden, 2005) and NO signaling (Crepel and Jaillard, 1990; Lev-Ram et al., 1995). The block of NO production by APV indicates that NMDARs are the trigger for NO production (Wang et al., 2014). However, the location of the NMDARs involved is a matter of debate. Since PCs do not express NMDARs at the developmental stages at which most plasticity experiments are conducted (Dupont et al., 1987) and because granule cells express NO synthase (Bredt et al., 1990), we proposed that the NMDARs involved in LTD are located on PFs. This idea was strongly supported by the observation of NMDAR subunits on PF varicosities contacting PC spines in electron immunohistochemistry (Bidoret et al., 2009). However, since MLIs express NMDARs and NO synthase, the signaling cascade has also been proposed to operate in MLI axons (Shin and Linden, 2005) or dendrites (Wang et al., 2014). Here, we found no functional evidence of axonal NMDARs in MLIs from juvenile rats. Moreover, we showed at both PF and CF connections that dendritic NMDARs are sensitive to GluN2B but not to GluN2A-specific pharmacology. Since NMDARs involved in PF-PC LTD presented a GluN2A-containing receptor pharmacology (LTD is prevented by Zn^2+^ but not by Ro25-6981; Bidoret et al., 2009), our results indicate that NMDARs on MLIs are not responsible for LTD at the PF-PC synapse.

## Author and contributors

CB, GB, GS and MC conceived and designed the experiments. CB, GB, AA and GS performed the experiments. CB, GB, AA, GS and MC analyzed the data and contributed to the writing of the manuscript.

## Conflict of interest statement

The authors declare that the research was conducted in the absence of any commercial or financial relationships that could be construed as a potential conflict of interest.
